# Ablação de Fibrilação Atrial: Eletroporação versus Ablação por Radiofrequência de Alta Potência e Curta Duração

**DOI:** 10.36660/abc.20240542

**Published:** 2025-03-12

**Authors:** Rita Reis Santos, Rita Amador, Pedro Galvão Santos, Daniel Matos, Gustavo Rodrigues, João Carmo, Francisco Costa, Pedro Carmo, Francisco Morgado, Diogo Cavaco, Mauricio Scanavacca, Pedro Adragão

**Affiliations:** 1 Centro Hospitalar de Lisboa Ocidental Hospital de Santa Cruz Lisboa Portugal Centro Hospitalar de Lisboa Ocidental Hospital de Santa Cruz, Carnaxide, Lisboa – Portugal; 2 Instituto do Coração do Hospital das Clínicas Faculdade de Medicina Universidade de São Paulo São Paulo SP Brasil Instituto do Coração do Hospital das Clínicas da Faculdade de Medicina da Universidade de São Paulo, São Paulo, SP – Brasil

**Keywords:** Fibrilação Atrial, Ablação por Radiofrequência, Veias Pulmonares

## Abstract

**Fundamento:**

O Isolamento da Veia Pulmonar (IVP) é crucial no tratamento de Fibrilação Atrial (FA). Novas tecnologias de ablação, tais como Ablação por Campo Pulsado (ACP) e ablação por radiofrequência de Alta Potência e Curta duração (HPSD, do inglês high-power short-duration) surgiram no laboratório de eletrofisiologia. No entanto, não há estudos comparando os desfechos dessas abordagens.

**Objetivo:**

Comparar a eficácia, a segurança da ACP e da ablação por HPSD em pacientes com FA sintomática.

**Métodos:**

Estudo retrospectivo, unicêntrico, de pacientes consecutivos submetidos ao IPP com ACP ou HPSD entre maio e dezembro de 2022. Foram analisados dados demográficos, dados relacionados ao procedimento, e recorrência de FA durante o seguimento. Foi realizada análise comparativa entre as duas técnicas. Um valor de p <0,05 foi considerado estatisticamente significativo.

**Resultados:**

Foram incluídos 101 pacientes (61±11 anos, 75% homens); 56% dos pacientes apresentaram FA paroxística e 19% foram submetidos a uma segunda ablação. Quarenta e cinco porcento dos pacientes foram submetidos à ablação por HPSD e 55% à ACP. Na comparação entre a ablação por HPSD e ACP, a primeira técnica apresentou um menor tempo de fluoroscopia [5min (IIQ 3-7min] vs. 13min (IQR 10-16min), p<0,001], porém um tempo de procedimento mais longo [97min (IQR 75-142) vs. 88min (IQR 66-111), p=0,13]. O Isolamento da Parede Posterior (IPP) foi realizado em cinco (11%) dos pacientes submetidos à ablação por HPSD vs. 20 (36%) dos submetidos à ACP (p=0,004). Houve somente um caso de complicação maior, um paciente com tamponamento cardíaco após a ACP, tratado com pericardiocentese. Ao longo do período de acompanhamento [384 (IIQ 341 -545) dias], 76 pacientes (75%) encontravam-se em ritmo sinusal, e 25% dos pacientes sofreram recorrência de FA (10 pacientes no grupo ACP e 15 no grupo HPSD, p=0,06).

**Conclusões:**

Observou-se que tanto a ACP como a ablação por HPSD é um procedimento viável e seguro. A ACP resultou em tempos mais curtos de procedimento e menores taxas de recorrência de FA, principalmente quando o IPP foi realizado. Embora avaliações no mundo real sejam ainda escassas, ambas as técnicas parecem eficientes, com uma baixa taxa de recorrência de FA.

## Graphical abstract

**Figura Central:**
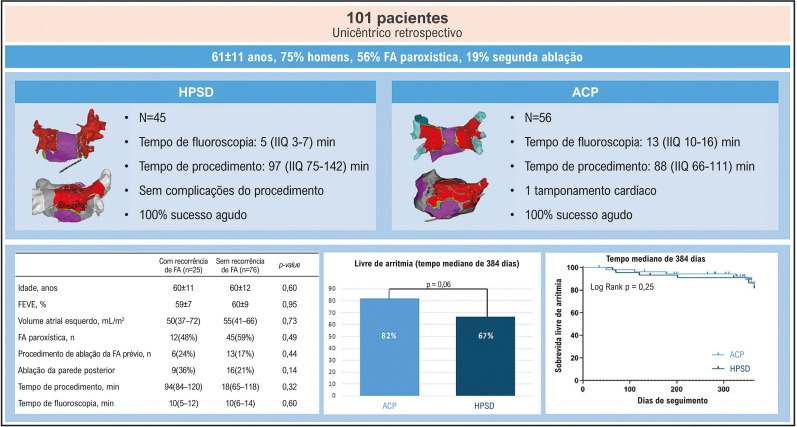
: Ablação de Fibrilação Atrial: Eletroporação versus Ablação por Radiofrequência de Alta Potência e Curta Duração

Ablação da fibrilação atrial por cateter: eletroporação versus ablação por radiofrequência de alta potência e curta duração.

## Introdução

A fibrilação atrial (FA) é a arritmia cardíaca mais comum, com um risco de ocorrência de cerca de 1 em 3-5 indivíduos com idade superior a 45 anos.^1^ Projeções apontam que, até o ano de 2050, a prevalência de FA aumentará para 15,9 milhões de pessoas na América e 17,9 milhões na Europa até 2060.^1^ A FA tem um grande impacto sobre a morbidade e a mortalidade, resultando em um risco aumentado de morte, insuficiência cardíaca, internações e eventos tromboembólicos.^1 , 2^

No tratamento de pacientes com FA sintomáticos, o isolamento da veia pulmonar (IVP) é um componente chave no controle de ritmo.^3^Novas tecnologias de ablação, tais como a ablação por radiofrequência de alta potência e curta duração (HPSD, do inglês *high-power short-duration* ) e a ablação por campo pulsado (ACP) surgiram recentemente de estudos eletrofisiológicos.

A ablação por cateter de arritmias atriais com HPSD surgiu como uma alternativa às modalidades convencionais, tipicamente realizadas com aplicação de energia de baixa potência. A ablação por HPSD visa produzir lesões mais superficiais, porém mais extensas, em um período mais curto reduzindo-se o aquecimento indireto (condutivo) e aumentando, simultaneamente, o aquecimento direto (resistivo).^4^ Isso pode resultar na formação mais efetiva de uma lesão superficial mais ampla, prevenindo-se, potencialmente, um dano colateral a estruturas adjacentes como o esôfago e os nervos frênicos.^5 , 6^

A ACP é uma técnica de energia avançada não termal que rompe as membranas celulares aplicando-se impulsos elétricos ultrarrápidos, resultando na formação irreversível de nanoporos e em apoptose celular.^7^ Por meio da otimização cuidadosa de parâmetros, tais como a amplitude de voltagem, formas de onda, e sequências de pulsos, a ACP pode seletivamente atingir o tecido miocárdico enquanto minimiza o dano a estruturas adjacentes.^8 , 9^

Os primeiros dados de longo prazo mostraram uma taxa de sobrevida em um ano livre de arritmia entre 70% e 85% para ambas as modalidades de ablação.^9 - 11^

Neste estudo original, avaliamos e comparamos a eficácia e a segurança de uma aplicação de ACP e HPSD para ablação da FA em pacientes sintomáticos.

## Métodos

### Dados clínicos, população e delineamento do estudo

Estudo retrospectivo unicêntrico incluindo pacientes com FA com idade inferior a 18 anos, encaminhados de maneira eletiva para ablação da FA sintomática em nosso centro terciário, entre maio e dezembro de 2022.

Foram excluídos do procedimento pacientes com FA secundária a desequilíbrio eletrolítico, doença tireoidiana, causa não cardíaca ou reversível, ou a trombo atrial esquerdo, e pacientes com contraindicação à anticoagulação. Parâmetros clínicos também foram coletados.

### Procedimento HPSD

Todos os pacientes foram submetidos à angiografia computadorizada cardíaca pré-FA para mapear as Veias Pulmonares (VPs) e excluir trombo no apêndice atrial esquerdo de acordo com o protocolo da instituição. Foi necessária terapia anticoagulação antes do procedimento, seguindo-se diretrizes atuais.^3^ Administrou-se heparina durante o procedimento de ablação para se atingir um tempo de coagulação ativado ≥ 325 segundos. Foi utilizado um novo cateter QDOT® para a ablação, em modo temperatura controlada, com taxas aumentadas de fluxo de irrigação e potências específicas para diferentes áreas do coração. Aplicações de Radiofrequência (RF) ponto a ponto foram utilizadas para criar um círculo contíguo ao redor das veias, sob temperatura e potência específicas (QMode+), com uma potência específica de 90W para a parede posterior por quatro segundos (fluxo de irrigação a 2-8 mL/min) e 50W para a parede anterior (fluxo de irrigação a 4-15 mL/min) com um índice de ablação alvo de 500-550.

Uma abordagem de sinais anatômicos e endocárdicos foi usada para isolar as VPs, e foi recomendado que aplicações de RF fossem feitas fora dos óstios das VPs para minimizar o risco de estenose. O IVP via bloqueio de entrada foi avaliado usando cateteres Lasso ou PentaRay (Biosense Webster, Inc., Irvine, CA). O tempo total do procedimento foi o período entre a obtenção do acesso vascular e a remoção dos cateteres do paciente.

Após o procedimento de ablação, os pacientes foram submetidos à terapia de anticoagulação oral por pelo menos dois meses. Após o período inicial, os pacientes foram orientados a continuarem a terapia de anticoagulação, de acordo com as diretrizes da ESC de 2020.^3^ A decisão de administrar drogas antiarrítmicas após a ablação foi feita pelo médico responsável pelo tratamento.

### Procedimento de ACP

Antes do procedimento, todos os pacientes foram submetidos à angiotomografia computadorizada para avaliar o átrio e as VPs e para excluir a presença de trombo no apêndice atrial esquerdo. Segundo diretrizes atuais,^3^ foi necessária terapia de anticoagulação sistêmica ininterrupta por pelo menos três semanas antes do tratamento.

Heparina não fracionada foi administrada antes da punção transseptal para manter um tempo de coagulação ativado entre 300 e 350 segundos.^12^ A ablação foi guiada por fluoroscopia, com ou sem um sistema de mapeamento eletroanatômico tridimensional (CARTO 3, Biosense Webster, Diamond Bar, CA, EUA). Os pacientes receberam sedação profunda com administração contínua de remifentanil e propofol ou anestesia geral, particularmente para ablação da parede posterior, segundo decisão médica.

Foi utilizado um cateter de multieletrodos tipo pentaspline (FARAPULSE, Boston Scientific) de ACP no procedimento. Outros cateteres foram posicionados no seio coronário e no ventrículo direito para controle do ritmo. O protocolo do grupo ACP envolveu o uso de cateter de ACP pentaspline (Farawave 12-Fr), inserido através de uma bainha manobrável com um eixo transparente (Faradrive) até o átrio esquerdo. Após posicionar o fio guia de ponta reta conforme recomendações (fio reto extra rígido Amplatz; Cook Inc.) em cada VP, o cateter de ACP foi posicionado no óstio de cada VP para administrar um total de oito lesões de campo pulsado por veia. Lesões adicionais foram realizadas em veias maiores ou no tronco pulmonar sempre que fossem detectados sinais anormais. As lesões foram distribuídas em um padrão de “cesta” e “flor”, com rotação entre cada par de lesões. Para ablação da parede posterior do átrio esquerdo, o cateter foi disposto em uma configuração de flor e posicionado ao longo da parede posterior do átrio esquerdo para administrar conjuntos sobrepostos de pulsos. O tempo total de procedimento foi o intervalo entre a obtenção do acesso vascular até a remoção dos cateteres do paciente. Como no grupo HPSD, após o procedimento de ablação, os pacientes foram submetidos à terapia de anticoagulação por pelo menos dois meses. Os pacientes foram orientados a continuar com a terapia anticoagulação seguindo-se as diretrizes da ESC,^3^ e a decisão de administrar drogas antiarrítmicas após a ablação foi feita pelo médico.

### Desfechos e acompanhamento

O desfecho primário foi sucesso agudo no IVP (e da parede posterior quando realizado). O desfecho secundário foi qualquer recorrência de arritmia atrial após um período de três meses. Recorrência foi caracterizada por palpitações com duração acima de 10 minutos ou detecção de FA, *flutter* atrial, ou qualquer arritmia atrial em eletrocardiograma (ECG) de rotina ou no Holter 24 horas.

O principal desfecho de segurança foi qualquer complicação maior relacionada ao procedimento, como tamponamento cardíaco, sangramento maior ou complicação vascular, infarto periprocedimento, paralisia persistente do nervo frênico, fístula antrioesofágica, e morte.

### Análise estatística

Todas as variáveis contínuas foram expressas em média ± Desvio Padrão (DP), ou mediana e Intervalo Interquartil (IIQ) para dados sem distribuição normal. A normalidade da distribuição dos dados foi verificada pelo teste de Kolmogorov-Smirnov. As variáveis categóricas foram expressas como números absolutos e porcentagens. Os grupos foram comparados usando o teste t de Student para amostras independentes quanto às variáveis contínuas com distribuição normal, o teste de Mann-Whitney para variáveis sem distribuição normal, e o teste exato de Fisher ou o teste qui-quadrado para variáveis categóricas. A significância estatística foi estabelecida em p<0,05 (bicaudal). As taxas de eventos cumulativos foram calculadas usando o método de Kaplan-Meier. O teste de log-rank foi usado para comparar a distribuição de eventos entre os dois grupos. Todas as análises foram realizadas usando o programa Statistical Package for the Social Sciences Statistics v27.0 (IBM Corporation, Armonk, NY, EUA).

## Resultados

### População do estudo e dados clínicos

Foram incluídos 101 pacientes consecutivos (75% homens), com idade média de 61±11 anos. A pontuação média no escore CHA2DS2-VASc foi 2±1 pontos; a fração de ejeção ventricular esquerda média foi 59± 8%, e o índice de volume atrial esquerdo mediano (tomografia computadorizada) foi 53 mL/m ^2^ [IIQ 40-58 mL/m^2^]. Cinquenta e seis porcento dos pacientes apresentavam FA paroxística, e 19% dos pacientes haviam se submetido a um procedimento de ablação. Em termos de fatores de risco cardiovasculares e comorbidades, 64 pacientes (63%) apresentavam um diagnóstico prévio de hipertensão, 41% apresentavam dislipidemia, e 17 pacientes (17%) eram diabéticos. Análises comparativas das características basais dos pacientes são apresentadas na Tabela 1 , indicando ausência de diferença estatisticamente significativa entre os dois grupos.

**Tabela 1:** – Características basais da coorte do estudo e análises comparativas entre os pacientes submetidos e à ablação por radiofrequência de alta potência e curta duração (HPSD, do inglês high-power short-duration) e os pacientes submetidos à ablação por campo pulsado (ACP)

Características basais	Total (n=101)	ACP (n=56)	HPSD (n=45)	Valor p
Idade, anos	61 ± 11	61±12	60±11	0,74
Sexo masculino	76 (75%)	35 (63%)	41 (91%)	0,01
IMC (kg/m^2^)	28±4	28±5	28±4	0,91
Hipertensão	64 (63%)	37 (66%)	27 (60%)	0,41
Diabetes mellitus	17(17%)	12 (21%)	5 (11%)	0,19
Dislipidemia	41(41%)	26 (46%)	15 (33%)	0,24
IM prévio	8 (8%)	4 (7%)	4 (9%)	0,72
AIT/AVC prévio	9 (9%)	5 (9%)	4 (9%)	0,70
Escore CHA2DS2-VASc	2±1	2±1	11	0,02
FA paroxística	57 (56%)	30 (54%)	27 (60%)	0,18
Procedimento de ablação da FA prévio	19 (19%)	12 (21%)	7 (16%)	0,45
**Ecocardiograma**				
FEVE	59 ± 8	59 ± 10	59 ± 5	0,81
**Tomografia computadorizada**				
Volume atrial esquerdo, mL/m^2^	53 (40-58)	56 (40-72)	51 (38-64)	0,47

Valores em mediana (intervalo interquartil), média ± desvio padrão; variáveis categóricas apresentadas como números absolutos e porcentagens; FA: fibrilação atrial; AIT: ataque isquêmico transitório; IM: infarto do miocárdio; AVC: acidente vascular cerebral; FEVE: fração de ejeção do ventrículo esquerdo.

### Resultados do procedimento de ablação da FA

Em relação à técnica de ablação da FA, 45% (n=45) dos pacientes realizaram HPSD e 55% (n=56) ACP ( Figura Central ). O mapeamento eletroanatômico foi conduzido em 94 (93%) pacientes (n=45 HPSD vs. n=49 ACP) com base na experiência inicial dos operadores e principalmente quando se planejou a realização de Isolamento da Parede Posterior (IPP). Em ambos os grupos, o IPP foi realizado com sucesso em todos (100%) os pacientes.

Pacientes com valores de volume atrial esquerdo mais altos eram mais propensos a serem submetidos ao IPP [63 mL/m^2^ (IIQ 42-76 mL/m^2^) vs. 48 mL/m^2^ [IQR 39-64 mL/m^2^], p=0,034). O IPP foi realizado principalmente nos pacientes submetidos à ACP. Os dados do procedimento estão apresentados na Tabela 2 . A duração do procedimento foi significativamente maior nos pacientes submetidos à ablação HPSD em comparação à ACP – 97 minutos (IIQ 75-142 min) vs. 88 minutos (IIQ 66-111 minutos), mas com tempos menores de fluoroscopia (5,4 min [IIQ 2,9-7,3 min] vs. 13,2 min [IIQ 10,2-15,6 min]). Em 13 pacientes (68%), houve necessidade de realização de outro IPP. Pacientes submetidos ao IPP apresentaram volumes de átrio esquerdo mais altos, embora sem significância estatística [62mL/m^2^ (IIQ 42-75 mL/m^2^) vs. 42 mL/m^2^ (IIQ 32-57 mL/m^2^), p=0.095].

**Tabela 2:** – Dados do procedimento de ablação e análises comparativas entre pacientes submetidos e à ablação por radiofrequência de alta potência e curta duração (HPSD, do inglês high-power short-duration) e os pacientes submetidos à ablação por campo pulsado (ACP)

Dados do procedimento de ablação da FA	Total (n=101)	ACP (n=56)	HPSD (n=45)	Valor p
Ablação da parede posterior	25 (25%)	20 (65%)	5 (11%)	0,004
Procedure time, minutos	91 (69 – 117)	88 (66 – 111)	97 (75 – 142)	0,13
Tempo de fluoroscopia, minutos	10 (5 – 14)	13 (10-16)	5 (3-7)	<0,001
Mapeamento eletroanatômico	94 (93%)	49 (88%)	45 (100%)	0,13
**Eventos adversos**				
Tamponamento pericárdico	1	1	-	-

Valores em mediana (intervalo interquartil), média ± desvio padrão; variáveis categóricas apresentadas como números absolutos e porcentagens.

Não foram relatadas complicações maiores para o grupo HSPD. No grupo ACP, não foram relatadas complicações, com exceção de um caso de tamponamento cardíaco com ACP, que foi tratado com pericardiocentese.

### Acompanhamento

**Figura 1:**
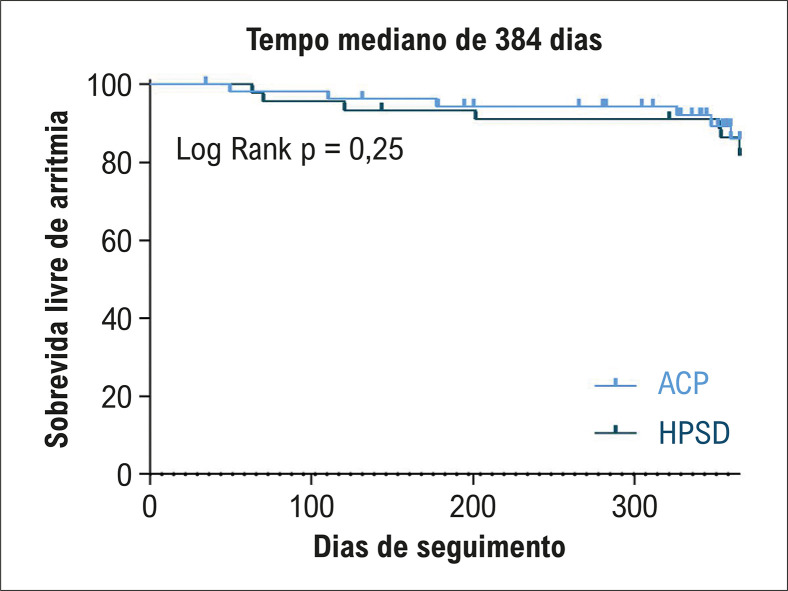
– Análise de Kaplan-Meier mostrando sobrevida livre de arritmia nos pacientes submetidos à ablação por radiofrequência de alta potência e curta duração (HPSD) e pacientes submetidos à ablação por campo pulsado (ACP).

Ao longo dos 384 dias (IIQ 341-545) e acompanhamento, o ECG realizado pelo menos três meses após a ablação da FA revelou que 76 pacientes (75%) estavam em ritmo sinusal, enquanto 25% dos pacientes apresentaram recorrência de FA – 10 pacientes no grupo ACP e 15 no grupo HPSD, 48% com FA paroxística. Apesar de não ter havido diferença estatística nas características basais nem nas características relacionadas ao procedimento entre os pacientes que sofreram recorrência de FA e os que não sofreram ( Tabela 3 ), os pacientes com recorrência de FA eram mais velhos (62 ±11 vs. 60 ± 12 anos), tinham maior chance de serem submetidos a um segundo procedimento (24 vs. 17%), e apresentaram maior porcentagem de IPP. Entre os pacientes submetidos ao IPP, a recorrência de FA foi observada em 80% dos indivíduos tratados com ablação por HPSD, e em 25% dos tratados com ACP (p=0,022). A análise de Kaplan-Meier ( Figura 1 ) não mostrou diferenças significativas na sobrevida livre de arritmia entre os dois grupos.

**Tabela 3:** – Análises comparativas entre pacientes com recorrência e sem recorrência de Fibrilação Atrial (FA) durante o seguimento

	Com FA recorrente (n=25)	Sem FA recorrente (n=76)	Valor p
Idade, anos	62 ± 11	60 ± 12	0,60
FEVE, %	59 ± 7	60 9	0,95
Volume atrial esquerdo, mL/m^2^	50 (37 – 72)	55 (41 – 66)	0,73
FA paroxística, n	12 (48%)	45 (59%)	0,49
Procedimento de ablação da FA prévio, n	6 (24%)	13 (17%)	0,44
Ablação da parede posterior	9 (36%)	16 (21%)	0,14
Tempo de procedimento, min	94 (84 – 120)	89 (65 – 118)	0,32
Tempo de fluoroscopia, min	10 (5 – 12)	10 (6 – 14)	0,60

Valores em mediana (intervalo interquartil), média ± desvio padrão; variáveis categóricas apresentadas como números absolutos e porcentagens; FA: fibrilação atrial; FEVE: fração de ejeção do ventrículo esquerdo.

A comparação dos pacientes com recorrência de FA ( Tabela 4 ) mostrou um tempo mais curto da fluoroscopia no grupo HPSD em comparação ao grupo ACP [5 minutos (IIQ 3-10 minutos) vs. 12 minutos (IIQ 10-18 minutos)], mas sem diferença estatisticamente significativa no tempo de procedimento [97 minutos (IIQ 90-156 minutos) vs. 90 minutos (IIQ 68-108 minutos)].

**Tabela 4:** – Análises comparativas entre pacientes submetidos à ablação por radiofrequência de Alta Potência e Curta duração (HPSD) e pacientes submetidos à Ablação por Campo Pulsado (ACP) com recorrência de FA

	ACP	HPSD	Valor p
Número de pacientes com recorrência de FA	10	15	0,06
Idade, anos	63 ± 9	61 ± 12	0,38
FEVE, %	59 ± 6	60 ± 9	0,69
Volume atrial esquerdo, mL/m^2^	49 (39 – 74)	53 (37 – 70)	0,89
FA paroxística, n	5 (50%)	7 (47%)	0,71
Procedimento de ablação da FA prévio, n	2 (20%)	4 (27%)	0,70
Ablação da parede posterior	5 (50%)	4 (27%)	0,23
Tempo de procedimento, min	90 (68 – 108)	97 (90 – 156)	0,34
Tempo de fluoroscopia, min	12 (10 – 18)	5 (3 – 10)	**<0,001**

Valores em mediana (intervalo interquartil), média ± desvio padrão; variáveis categóricas apresentadas como números absolutos e porcentagens; FA: fibrilação atrial; FEVE: fração de ejeção do ventrículo esquerdo.

Análises de subgrupos de pacientes com FA que sofreram recorrência de FA durante o acompanhamento indicaram ausência de diferenças estatisticamente significativas entre os pacientes com e sem FA paroxística ( Tabela Suplementar 1 ). Além disso, não se observou diferença no tipo de FA entre os grupos HPSD e ACP ( Tabelas Suplementares 2 e 3 ). Ainda, não foram observadas diferenças significativas entre os pacientes com FA recorrente e submetidos à ablação da parede posterior no grupo HPSD e no grupo ACP ( Tabela Suplementar 4 ).

## Discussão

Este estudo compara nossa experiência inicial com novas tecnologias de ablação: ACP e HPSD. Com base em nosso conhecimento atual, há pouca evidência comparando os procedimentos de HPSD e ACP, principalmente a respeito de complicações do procedimento e dados longitudinais após a intervenção. Nosso estudo avalia nossa experiência inicial com essas técnicas inovadoras, abordando os procedimentos, complicações relacionadas e acompanhamento após a intervenção.

Este estudo inclui dois grupos de pacientes sem diferenças estatisticamente diferentes em suas características basais. Um IVP bem-sucedido é um fator chave na eficácia da ablação. Em concordância com resultados previamente relatados da ACP e da ablação de HPSD,^13 , 14^ nós alcançamos 100% de sucesso do procedimento em termos técnicos.

De acordo com nossos resultados, e consistente com registros prévios, tais como os relatados no ensaio MANIFEST,^15^ a ACP demonstrou uma nítida vantagem em termos de um tempo de procedimento mais curto. A redução no tempo de procedimento minimiza a exposição do paciente a agentes anestésicos, fluidos intravenosos e heparina, enquanto melhora significativamente a eficiência do procedimento.

Consistente com nossos dados, ensaios prévios relataram tempos mais curtos de fluoroscopia para HPSD em comparação à ACP.^11^ Como a ACP é uma técnica mais recente em comparação à ablação de HPSD, um fator que contribui para os tempos mais longos de fluoroscopia está provavelmente relacionado ao fenômeno de curva de aprendizagem, já que operadores mais experientes tendem a realizar o procedimento de maneira mais eficiente e com um uso relativamente menor de fluoroscopia. Contudo, apesar da expertise dos operadores e do mapeamento eletroanatômico, a ACP requer fluoroscopia para confirmar o posicionamento e a rotação do cateter. Tal fato resulta em um tempo de fluoroscopia um pouco mais longo em comparação às técnicas de ablação que não requerem confirmação radiográfica.

Outro ponto chave é o fato de que ambos os grupos apresentaram poucas complicações do procedimento, em consonância com dados prévios para ambos os grupos.^4 , 15^ Não foram relatadas complicações no grupo HPSD e houve somente um caso de tamponamento cardíaco no grupo ACP. Neste paciente, a causa do tamponamento cardíaco não foi identificada facilmente; no entanto, ao final do procedimento de ACP, o cateter Farawave^TM^ foi removido da bainha Faradrive^TM^, e o átrio esquerdo remapeado com um cateter Pentaray®. Assim, a manipulação do cateter pode ter sido a causa do tamponamento cardíaco, que foi tratado com pericardiocentese, sem necessidade de cirurgia.

Similarmente a estudos prévios,^7 , 10 , 16^ uma alta porcentagem de pacientes permaneceram livre de FA no seguimento em curto prazo, com uma taxa de recorrência mais baixa no grupo ACP e um número estatisticamente menor de recorrências nos pacientes submetidos ao IPP no grupo ACP em comparação ao grupo HPSD. Uma vez que nossa coorte não exibiu diferenças estatisticamente significativas quanto às características basais entre os pacientes submetidos ao IPP no grupo ACP e HPSD, atribuímos a diferença na eficácia em longo prazo à técnica de ablação empregada, particularmente à homogeneidade das lesões da ablação geradas pela ACP.

Nesse contexto, a ACP foi a técnica preferida para o IPP planejado, demonstrando resultados excelentes do procedimento, atestando, assim, sua facilidade e efetividade. Vale ressaltar que o IPP foi conduzido com base na descrição do operador e no volume atrial esquerdo, e guiado principalmente pela avaliação dos potenciais fragmentados e/ou fibrose atrial.

Por fim, em nosso estudo, em relação à recorrência de FA, não foram observadas diferenças estatisticamente significativas entre os vários subgrupos analisados. O único fator de diferenciação foi a técnica de ablação empregada, com uma tendencia a melhores desfechos com ACP, especialmente quando o IPP foi realizado.

Este estudo está sujeito a várias limitações importantes que devem ser consideradas na interpretação dos achados. Primeiro, o estudo foi conduzido em um único centro terciário com um tamanho amostral moderado. Tal fato limita a extrapolação dos resultados a outros ambientes ou populações. Além disso, o período mediano de acompanhamento de aproximadamente um ano é relativamente curto para avaliar desfechos em longo prazo, tal como recorrência de FA. Assim, é necessário um período mais longo de acompanhamento para avaliar a duração dos efeitos da ablação. Segundo, este estudo se trata de uma análise retrospectiva, não randomizada. Embora ele inclua dois grupos de pacientes que não apresentavam características basais estatisticamente significativas, a falta de randomização pode haver introduzido um viés de seleção. Estudos retrospectivos dependem de dados pré-existentes e registos médicos, os quais podem introduzir vieses ou inconsistências na coleta de dados. Além disso, este estudo não considerou informações detalhadas sobre terapias antiarrítmicas, o que poderia influenciar substancialmente os desfechos do manejo da FA e confundir as associações relacionadas à recorrência de FA.

Em resumo, enquanto este estudo contribui com *insights* valiosos, suas limitações destacam a necessidade de mais estudos com populações maiores e delineamento prospectivo para validar e detalhar esses resultados.

## Conclusão

Tanto a ACP e a ablação por HPSD são opções seguras e viáveis, com uma tendência a uma eficácia superior da ACP. Relatamos um caso de tamponamento cardíaco no grupo ACP, que ocorreu durante um procedimento realizado quando se havia pouca experiência no centro e atribuído à manipulação do cateter.

O tempo de procedimento foi mais curto com ACP e, embora não estatisticamente significativo, esse fato foi associado com uma taxa mais baixa de recorrência de FA em comparação à ablação HPSD. Especificamente, o grupo ACP exibiu taxas significativamente mais baixas de recorrência de FA quando o IPP foi realizado, em contraste com o grupo tratado com ablação por HPSD. Avaliações de ambas as técnicas ainda são poucas no mundo real, mas os dois métodos foram eficazes em temos de baixa recorrência de FA durante o seguimento.
