# Supplementary material for: Ablação de Fibrilação Atrial: Eletroporação versus Ablação por Radiofrequência de Alta Potência e Curta Duração
Source: Arq Bras Cardiol. 2025 Mar 12;122(2):e20240542. [Article in Portuguese] doi: 10.36660/abc.20240542 (PMC12080718; doi:10.36660/abc.20240542)
Supplement: Supplementary file 1 [file 0066-782X-abc-122-2-e20240542-suppl01.pdf]

Supplemental Table 1 – Comparative analyses between paroxysmal and non-paroxysmal atrial fibrillation (AF) patients with AF recurrence on follow-up

	**Paroxysmal AF (n=12)**	**Non- paroxysmal AF (n=13)**	***p-value***
Age, years	61 ± 13	62 ± 9	0.81
LV ejection fraction, %	58 ± 10	60 ± 3	0.52
Left atrial volume index, mL/m2	56 (36 – 75)	48 (38 – 71)	0.83
Previous AF-ablation procedure, n	1 (8%)	5 (39%)	0.08
Posterior Wall ablation, n	3 (25%)	6 (46%)	0.27
Procedure time, min	93 (88 – 124)	97 (74 – 132)	0.83
Fluoroscopy time, min	8 (4 – 13)	8 (5 – 11)	0.92

Values are median (interquartile range), mean±standard deviation. Categorical variables are presented as absolute numbers (and percentages). AF- atrial fibrillation; LV – left ventricle.

Supplemental Table 2 – Comparative analyses between patients with atrial fibrillation (AF) recurrence and paroxysmal AF in HPSD and PFA groups

	**PFA (n=5)**	**HPSD (n=7)**	***p-value***
Age, years	65±17	64±8	0.69
LV ejection fraction, %	55±6	60±13	0.55
Left atrial volume index, mL/m2	67 (54 – 72)	62 (36 – 70)	0.76
Posterior wall ablation, n	1 (20%)	2 (29%)	0.74
Previous AF-ablation procedure, n	0 (0%)	1 (14%)	0.37
Procedure time, min	92 (88 – 111)	92 (89 – 97)	0.88
Fluoroscopy time, min	13 (11 – 13)	7 (3 – 10)	0.30

Values are median (interquartile range), mean±standard deviation. Categorical variables are presented as absolute numbers (and percentages). AF- atrial fibrillation; LV – left ventricle.

Supplemental Table 3 – Comparative analyses between patients with atrial fibrillation (AF) recurrence and non-paroxysmal AF in HPSD and PFA groups

	PFA (n=5)	HPSD (n=8)	*p-value*
Age, years	61±5	60±12	0.72
LV ejection fraction, %	61±5	60±1	0.68
Left atrial volume index, mL/m2	48 (42 – 73)	38 (35 – 52)	0.83
Posterior wall ablation, n	4 (80%)	2 (25%)	0.53
Previous AF-ablation procedure, n	2 (32%)	3 (38%)	0.93
Procedure time, min	79 (69 – 100)	94 (77 – 100)	0.28
Fluoroscopy time, min	11 (11 – 18)	5 (4 – 6)	0.30

Values are median (interquartile range), mean±standard deviation. Categorical variables are presented as absolute numbers (and percentages). AF- atrial fibrillation; LV – left ventricle.

Supplemental Table 4 – Comparative analyses between patients with atrial fibrillation (AF) recurrence and posterior wall ablation in HPSD and PFA groups.

	PFA (n=5)	HPSD (n=4)	*p-value*
Age, years	62 ± 6	59 ± 8	0.52
LV ejection fraction, %	62 ± 5	60 ± 18	0.82
Left atrial volume index, mL/m2	48 (36 – 63)	39 (35 – 50)	0.54
Paroxysmal AF, n	1 (20%)	2 (50%)	0.34
Previous AF-ablation procedure, n	2 (40%)	3 (75%)	0.29
Procedure time, min	100 (74 – 109)	91 (89 – 150)	0.81
Fluoroscopy time, min	11 (9 – 18)	9 (3 – 10)	0.30

Values are median (interquartile range), mean±standard deviation. Categorical variables are presented as absolute numbers (and percentages). AF- atrial fibrillation; LV – left ventricle.
