# Supplementary material for: Ablação de Fibrilação Atrial: Eletroporação versus Ablação por Radiofrequência de Alta Potência e Curta Duração
Source: Arq Bras Cardiol. 2025 Mar 12;122(2):e20240542. [Article in Portuguese] doi: 10.36660/abc.20240542 (PMC12080718; doi:10.36660/abc.20240542)
Supplement: Translation

# Translation: Atrial Fibrillation Catheter Ablation: Electroporation Against High-Power Short Duration Radiofrequency

**DOI:** 10.36660/abc.20240542i

**Authors:** Rita Reis Santos, Rita Amador, Pedro Galvão Santos, Daniel Matos, Gustavo Rodrigues, João Carmo, Francisco Costa, Pedro Carmo, Francisco Morgado, Diogo Cavaco, Mauricio Scanavacca, Pedro Adragão

**Affiliations:** 1 Portugal Centro Hospitalar de Lisboa Ocidental Hospital de Santa Cruz, Carnaxide, Lisboa – Portugal; 2 Brasil Instituto do Coração do Hospital das Clínicas da Faculdade de Medicina da Universidade de São Paulo, São Paulo, SP – Brasil

**Keywords:** Atrial Fibrillation, Radiofrequency Ablation, Pulmonary Veins

## Abstract

**Background:**

Pulmonary vein isolation (PVI) is crucial in treating symptomatic atrial fibrillation (AF). New ablation technologies, such as pulse-field ablation (PFA) and high-power short-duration (HPSD) have emerged in the electrophysiology lab. However, no study has compared the outcomes of these approaches.

**Objective:**

To compare the efficacy and safety of PFA and HPSD in AF symptomatic patients.

**Methods:**

Single-centre, retrospective study of consecutive patients undergoing PVI with PFA or HPSD between May and December 2022. Demographic data, procedural data, and AF recurrence beyond the blanking period, were analysed. Comparative analysis between both techniques was performed. A P-value of <0.05 was considered statistically significant.

**Results:**

A total 101 patients were included (61±11 years, 75% men); 56% of patients had paroxysmal AF and 19% underwent a redo ablation. Forty-five percent of patients underwent HPSD ablation and 55% PFA. Comparing HPSD and PFA, HPSD had a lower fluoroscopy time (5min [IQR 3-7min]vs 13min [IQR 10-16min], p<0.001), but higher procedure time (97min [IQR 75-142]vs 88min [IQR 66-111], p=0.13). Posterior wall isolation (PWI) was performed in 5 (11%) HPSD vs 20 (36%) PFA patients (p=0.004). There was only one case of major complication, a patient with cardiac tamponade following PFA, who was treated with pericardiocentesis. Over 384 (IQR 341 -545) days of follow-up, 76 patients (75%) were in sinus rhythm, while 25% of patients had AF recurrence: 10 PFA patients and 15 HPSD patients (p=0.06).

**Conclusions:**

Both PFA and HPSD were found to be feasible and safe procedures. PFA resulted in shorter procedure times, and lower AF recurrence rates, mainly when PWI was performed. Although analysis in a real-world scenario is still scarce, both techniques seem to be efficient, with a low AF recurrence rate.

## Introduction

Atrial fibrillation (AF) is the most frequent cardiac arrhythmia with a lifetime risk of about 1 in 3–5 individuals after the age of 45 years.^1^Projections indicate that by 2050, the prevalence of AF will rise to 15.9 million in America and 17.9 million in Europe by 2060.^1^AF has a profound impact on global morbidity and mortality, resulting in an increased risk of death, heart failure, hospital admissions, and thromboembolic events.^1 , 2^

When treating symptomatic AF patients, pulmonary vein isolation (PVI) is a key component of rhythm-control therapy.^3^New ablation technologies, such as high-power short-duration (HPSD) radiofrequency ablation and pulsed-field ablation (PFA), have been recently introduced in the electrophysiology (EP) lab.

Catheter ablation of atrial arrhythmias with HPSD emerged as an alternative to conventional ablation modes, typically performed at low-power energy settings. HPSD ablation aims to produce shallower yet broader lesions in a short duration by reducing indirect (conductive) heating while simultaneously increasing direct (resistive) heating.^4^This may result in more effective, broader, and superficial lesion formation potentially avoiding collateral damage to adjacent structures like the oesophagus and the phrenic nerves.^5 , 6^

PFA is an advanced nonthermal energy technique that disrupts cell membranes by applying ultra-rapid electrical impulses, resulting in irreversible nanoscale pore formation, and cellular apoptosis.^7^Through the careful optimization of parameters such as voltage amplitude, waveforms, and pulse sequences, PFA can selectively target myocardial tissue while minimizing damage to adjacent structures.^8 , 9^

First long-term data reported an arrhythmia-free survival after one year reaching from 70% to 85% for both AF ablation modalities.^9 - 11^

In this original research work, we evaluated and compared the efficacy and safety of single-shot PFA and HPSD for AF ablation in symptomatic patients.

## Methods

### Clinical data, and study population and design

Single-centre retrospective study including patients with AF, under 18 years of age, and electively referred for symptomatic AF ablation in our tertiary centre, between May and December 2022.

Patients were excluded from the procedure if they had AF secondary to electrolyte imbalance, thyroid disease, reversible or non-cardiac cause, or left atrial thrombus, and patients with contraindications to anticoagulation. Clinical parameters were also collected.

### HPSD procedure

All patients underwent pre-AF ablation cardiac computed angiography to map the pulmonary veins (PVs) and rule out left atrial appendage thrombus following the institutional protocol. Anticoagulation therapy was required before the procedure in accordance with current guidelines.^3^Heparin was administered during the ablation procedure to achieve an activated clotting time of ≥325 seconds. A novel QDOT® catheter in temperature-controlled mode was used for the ablation, with increased irrigation flow rates and specific power settings for different areas of the heart. Point-by-point radiofrequency (RF) delivery was used to create a contiguous circle around the veins, with specific temperature and power (QMode+), with a power of 90W for the posterior wall for four seconds (irrigation flow at 2-8 mL/min) and 50W for the anterior wall (irrigation flow 4-15 ml/min) with a target ablation index of 500-550.

An anatomical and endocardial signals approach was used to isolate all PVs, and RF applications were recommended to be outside the PV ostia to minimize the risk of PV stenosis. PVI via the entrance block was assessed using Lasso or PentaRay catheters (Biosense Webster, Inc., Irvine, CA). The total procedure time was the interval from obtaining vascular access to removing catheters from the patient.

After the ablation procedure, patients underwent oral anticoagulation therapy for at least two months. Following this initial period, patients were advised to continue anticoagulation therapy in accordance with the 2020 ESC guidelines.^3^The decision to administer antiarrhythmic drugs after the ablation was left to the treating physician’s discretion.

### PFA procedure

Prior to the procedure, all patients underwent cardiac computed tomography angiography to assess the atrium and PVs and to rule out the presence of left atrial appendage thrombus. According to current guidelines,^3^uninterrupted systemic anticoagulation therapy was required for at least three weeks before treatment.

Unfractionated heparin was given before the transseptal puncture to maintain an activated clotting time between 300 and 350 seconds.^12^The ablation was performed under fluoroscopic guidance, with or without a three-dimensional electroanatomic mapping system (CARTO 3, Biosense Webster, Diamond Bar, CA, USA). Patients received deep sedation using continuous remifentanil and propofol infusion or general anaesthesia, particularly for posterior wall ablation, according to the physician’s discretion.

The multielectrode pentaspline PFA catheter (FARAPULSE, Boston Scientific) was utilized for ablation. Other catheters were positioned in the coronary sinus and right ventricle for pacing if needed. The PFA group protocol involved using a pentaspline PFA catheter (Farawave, 12-Fr) inserted through a 13-Fr steerable sheath with a transparent shaft (Faradrive) into the left atrium (LA). After placing the recommended straight-tip 0.035 guidewire (Amplatz extra stiff straight wire; Cook Inc.) in each target PV, the PFA catheter was placed at the ostium of each PV to administer a total of eight pulsed-filed (PF) lesions per vein. Additional lesions were performed on larger veins or the pulmonary trunk whenever abnormal signals were detected. The lesions were distributed in a ‘basket’ and ‘flower’ pattern, with rotation between each pair of lesions. For ablating the posterior LA wall, the catheter was deployed in a flower configuration and placed along the posterior LA to deliver overlapping sets of pulses. The total procedure time was the interval from obtaining vascular access to the removal of catheters from the patient. Like in HPSD group, after the ablation procedure, patients underwent oral anticoagulation therapy for at least two months. They were then advised to continue anticoagulation therapy as per the 2020 ESC guidelines^3^and the decision to administer antiarrhythmic drugs post-ablation was made by the physician.

### Outcomes and follow-up

The primary outcome was acute success in PVI (and posterior wall when performed). The secondary outcome was the presence of any recurrence of atrial arrhythmia after a 3-month blanking period. Recurrence was characterised by palpitations lasting over 10 minutes or the detection of AF, atrial flutter, or any atrial arrhythmia on a routine electrocardiogram (ECG) or on 24-hour Holter monitoring.

The main safety outcome was any procedure-related major complications (e.g., cardiac tamponade, major bleeding or vascular complication, peri-procedural stroke, persistent phrenic nerve palsy, atrioesophageal fistula, and death).

### Statistical analysis

All continuous variables were expressed as mean ± standard deviation (SD), or median and interquartile range (IQR) for skewed data. Normality of data distribution was checked using the Kolmogorov-Smirnov test. Categorical variables were expressed as absolute numbers and percentages. Groups were compared using the independent-samples Student’s t-test for normally distributed continuous variables, the Mann- Whitney U test for non-normally distributed variables, and the Fisher exact test or a chi-square test for categorical variables. Statistical significance was set at P-value <0.05 (two-sided). Cumulative event rates were calculated using the Kaplan–Meier method. A log-rank test was performed to compare event distribution between both groups. All analyses were performed using Statistical Package for the Social Sciences Statistics v27.0 (IBM Corporation, Armonk, NY, USA).

## Results

### Study population and clinical data

A total of 101 consecutive patients (75% men) were included in the study, with a mean age of 61 ± 11 years. The mean CHA2DS2-VASc score was 2±1 points; mean left ventricular ejection fraction was 59± 8%, and median left atrial volume index (by computed tomography scan) was 53 mL/m^2^[IQR 40-58 mL/m^2^]. Fifty-six percent of the patients had paroxysmal AF, and 19% had performed a previous ablation procedure. In terms of cardiovascular risk factors and comorbidities, 64 patients (63%) had a previous diagnosis of hypertension, 41% had dyslipidaemia, and 17 patients (17%) were diabetic. Comparative analyses of the baseline characteristics of the patients are shown in Table 1 , indicating no statistically significant differences between the two groups.

**Table 1:** – Baseline characteristics of the study cohort and comparative analyses between patients undergoing high-power short-duration (HPSD) radiofrequency ablation and those undergoing pulsed-field ablation (PFA)

Baseline characteristics	Total (n=101)	PFA (n=56)	HPSD (n=45)	p-value
Age, years	61 ± 11	61±12	60±11	0.74
Male	76 (75%)	35 (63%)	41 (91%)	0.01
BMI (kg/m^2^)	28±4	28±5	28±4	0.91
Hypertension	64 (63%)	37 (66%)	27 (60%)	0.41
Diabetes mellitus	17(17%)	12 (21%)	5 (11%)	0.19
Dyslipidemia	41(41%)	26 (46%)	15 (33%)	0.24
Previous MI	8 (8%)	4 (7%)	4 (9%)	0.72
Previous TIA/stroke	9 (9%)	5 (9%)	4 (9%)	0.70
CHA2DS2-VASc, points	2±1	2±1	11	0.02
Paroxysmal AF	57 (56%)	30 (54%)	27 (60%)	0.18
Previous AF-ablation procedure	19 (19%)	12 (21%)	7 (16%)	0.45
**Echocardiography**				
LV ejection fraction	59 ± 8	59 ± 10	59 ± 5	0.81
**Computed Tomography scan**				
Left atrial volume index, mL/m^2^	53 (40-58)	56 (40-72)	51 (38-64)	0.47

Values are median (interquartile range), mean±standard deviation. Categorical variables are presented as absolute numbers (and percentages). AF: atrial fibrillation; MI: myocardial infarction; TIA: transient ischemic attack; LV: left ventricular.

### AF-ablation procedure results

Regarding AF ablation technique, 45% (n=45) of patients performed HPSD and 55% (n=56) PFA ( Central Illustration ). Electroanatomical mapping was performed in 94 (93%) patients (n=45 HPSD vs. n=49 PFA) based on the initial experience of the operators and, mainly, when posterior wall isolation (PWI) was planned. In both groups, PVI was successfully performed in all patients (100%).

Patients with larger left atrial volume indexes were more likely to undergo PWI (63 mL/m^2^[IQR 42-76 mL/m^2^] vs. 48 mL/m^2^[IQR 39-64 mL/m^2^], p=0.034). PWI was performed mostly in PFA patients – 20 (65%) vs 5 (11%). Procedural data are displayed in Table 2 . Procedure duration was significantly longer in HPSD patients as compared to PFA (97 min [IQR 75-142 min] vs. 88 min [IQR 66-111 min]), although with shorter fluoroscopy times (5.4 min [IQR 2.9-7.3 min] vs 13.2 min [IQR 10.2-15.6 min]). Considering the redo procedures, in 13 patients (68%) a PWI was performed. Although not statistically significant, patients in whom PWI was performed had larger LA volume indexes (62mL/m^2^[IQR 42-75 mL/m^2^] vs 42 mL/m^2^[IQR 32-57 mL/m^2^], p=0.095).

**Table 2:** – Ablation procedure data and comparative analyses between patients undergoing high-power short-duration (HPSD) radiofrequency ablation and those undergoing pulsed-field ablation (PFA)

AF ablation procedure data	Total (n=101)	PFA (n=56)	HPSD (n=45)	p-value
Posterior wall ablation	25 (25%)	20 (65%)	5 (11%)	0.004
Procedure time, min	91 (69 – 117)	88 (66 – 111)	97 (75 – 142)	0.13
Fluoroscopy time, min	10 (5 – 14)	13 (10-16)	5 (3-7)	<0.001
Electroanatomical mapping	94 (93%)	49 (88%)	45 (100%)	0.13
**Adverse Events**				
Pericardial tamponade	1	1	-	-

Values are median (interquartile range), mean±standard deviation. Categorical variables are presented as absolute numbers (and percentages); AF: atrial fibrillation.

No major complications were reported for the HPSD group. In the PFA group, no other complications were reported, aside from a cardiac tamponade with PFA which was treated with pericardiocentesis.

### Follow-up

Over 384 [IQR 341 – 545] days of follow-up, ECG performed at least three months after AF ablation revealed that 76 patients (75%) were in sinus rhythm, while 25% of patients had AF recurrence: 10 PFA patients and 15 HPSD patients, 48% with paroxysmal AF. Although baseline and procedural characteristics showed no significant differences between the groups that experienced a relapse and those that did not ( Table 3 ), patients with AF recurrence were older (62 ±11 vs. 60 ± 12 years), were more likely to undergo a redo procedure (24 vs. 17%), and had a higher percentage of PWI (36% vs. 21%). Among patients who underwent PWI, AF recurrence was observed in 80% of those treated with HPSD ablation compared to 25% of those treated with PFA (p=0.022). Kaplan-Meier analysis ( Figure 1 ) showed no significant differences in arrhythmia-free survival between both groups.

**Table 3:** – Comparative analyses between patients with atrial fibrillation recurrence on follow-up and no AF recurrence

	AF recurrence (n=25)	No AF-recurrence (n=76)	p-value
Age, years	62 ± 11	60 ± 12	0.60
LV ejection fraction, %	59 ± 7	60 9	0.95
Left atrial volume index, mL/m^2^	50 (37 – 72)	55 (41 – 66)	0.73
Paroxysmal AF, n	12 (48%)	45 (59%)	0.49
Previous AF-ablation procedure, n	6 (24%)	13 (17%)	0.44
Posterior wall ablation	9 (36%)	16 (21%)	0.14
Procedure time, min	94 (84 – 120)	89 (65 – 118)	0.32
Fluoroscopy time, min	10 (5 – 12)	10 (6 – 14)	0.60

Values are median (interquartile range), mean±standard deviation. Categorical variables are presented as absolute numbers (and percentages). AF: atrial fibrillation; LV: left ventricular.

**Figure 1:** – Kaplan-Meier analysis showing arrhythmia-free survival during follow-up in patients undergoing high-power short-duration (HPSD) radiofrequency ablation and those undergoing pulsed-field ablation (PFA).

The comparison of patients with AF recurrence ( Table 4 ) revealed a shorter fluoroscopy time in the HPSD group compared to PFA (5 min [IQR 3-10 min] vs. 12 min [IQR 10-18 min]), but no statistical difference in procedure times (97 min [IQR 90-156 min] vs. 90 min [IQR 68-108 min]).

**Table 4:** – Comparative analyses between patients with atrial fibrillation recurrence on follow-up and in patients undergoing high-power short-duration (HPSD) radiofrequency ablation and those undergoing pulsed-field ablation (PFA)

	PFA	HPSD	p-value
Number of patients with AF-recurrence	10	15	0.06
Age, years	63 ± 9	61 ± 12	0.38
LV ejection fraction, %	59 ± 6	60 ± 9	0.69
Left atrial volume index, mL/m^2^	49 (39 – 74)	53 (37 – 70)	0.89
Paroxysmal AF, n	5 (50%)	7 (47%)	0.71
Previous AF-ablation procedure, n	2 (20%)	4 (27%)	0.70
Posterior Wall ablation, n	5 (50%)	4 (27%)	0.23
Procedure time, min	90 (68 – 108)	97 (90 – 156)	0.34
Fluoroscopy time, min	12 (10 – 18)	5 (3 – 10)	<0.001

Values are median (interquartile range), mean±standard deviation. Categorical variables are presented as absolute numbers (and percentages). AF: atrial fibrillation; LV: left ventricular.

Subgroup analyses of patients with AF who experienced AF recurrence during follow-up indicated no statistically significant differences between those with paroxysmal AF and those with non-paroxysmal AF ( Supplementary Table 1 ). Additionally, no significant differences were observed in AF type between the HPSD and PFA groups ( Supplementary Tables 2 and 3 ).

Likewise, no statistically significant differences were observed between patients with AF recurrence who underwent posterior wall ablation in HPSD and PFA groups ( Supplementary Table 4 ).

## Discussion

This study compares our initial experience with new ablation technologies: PFA and HPSD. Based on our current knowledge, evidence is scarce comparing HPSD and PFA procedures, mainly concerning procedure complications and longitudinal data following the intervention. Our study evaluates our initial experience with these innovative techniques, addressing the procedures, related complications, and post-intervention follow-up.

This study includes two groups of patients with no statistically significant differences in their basal characteristics. Successful PVI is a key factor in the efficacy of ablation. In line with some previously reported PFA and HPSD results,^13 , 14^we achieved 100% intraprocedural technical success.

According to our results, and consistent with previous registries, such as the MANIFEST trial,^15^PFA demonstrated a clear advantage in terms of shorter procedure time. The reduction in procedure time minimizes patient exposure to anaesthetic agents, intravenous fluids and heparin, while significantly enhancing procedural efficiency.

Consistent with our data, previous trials reported shorter fluoroscopy times for HPSD compared to PFA.^11^Since PFA is a more recent technique compared to HPSD, a contributory factor for the higher fluoroscopy times is likely related to the learning curve phenomenon, as more experienced operators tend to perform the procedure more efficiently and with a relatively lower use of fluoroscopy. However, despite operators’ expertise and electroanatomic mapping, PFA requires fluoroscopy to confirm catheter rotation and positioning. This requirement results in a slightly longer fluoroscopy time compared to other ablation techniques that do not require radiographic confirmation.

Another key point is that both groups showed very few procedural complications, in line with previous data for both groups.^4 , 15^No complications were reported in the HPSD group and only one case of cardiac tamponade in the PFA group was reported. In this patient, the cause of cardiac tamponade was not easily identified; however, at the end of the PFA procedure, the Farawave^TM^catheter was removed from the Faradrive^TM^sheath and the left atrium was remapped with a Pentaray® catheter after ablation. Thus, catheter manipulation may have caused cardiac tamponade, which was managed with pericardiocentesis, without the need for cardiac surgery.

In line with previous reports,^7 , 10 , 16^a high percentage of patients remained free from AF in the short-term follow-up, with a lower AF recurrence in the PFA group and statically fewer recurrences when PWI was performed in PFA compared to HPSD. Given that our cohort did not exhibit statistically significant differences in baseline characteristics between patients undergoing PWI in PFA and HPSD, we attribute the observed difference in long-term efficacy to the specific ablation technique employed, particularly the homogeneity of the ablation lesions generated by PFA.

In this context, PFA was the preferred technique for planned PWI, demonstrating excellent intraprocedural results, thereby attesting to its ease and effectiveness. It is noteworthy that PWI was conducted based on the operator’s description and on LA volume and primarily guided by the assessment of fragmented potentials and/or LA fibrosis.

Finally, in our study, concerning AF recurrence, no statistically significant differences were observed across the various subgroups analysed. The sole differentiating factor was the ablation technique employed, with a tendency towards better outcomes with PFA, mainly when PWI was performed.

This study is subject to several important limitations that should be considered when interpreting its findings. Firstly, it was conducted at a single tertiary centre with a moderate sample size, which limits the extrapolation of the findings to other settings or populations. Additionally, the median follow-up period of approximately one year is relatively brief for evaluating long-term outcomes, such as AF recurrence. Thus, a longer follow-up period to fully assess the duration of the ablation effects is needed. Second, this study is a non-randomized, retrospective analysis. Although it includes two groups of patients with no statistically significant differences in their basal characteristics, the lack of randomization could introduce selection bias. Retrospective studies inherently rely on pre-existing data and medical records, which may introduce biases or inconsistencies in data collection. Furthermore, this study did not capture detailed information on antiarrhythmic therapies, which could substantially influence AF management outcomes and confound the associations related to AF recurrence.

In summary, while this study contributes valuable insights, its limitations underscore the need for further research with larger populations and prospective study designs to validate and expand upon these findings.

## Conclusion

Both PFA and HPSD were found to be feasible and safe options, with a trend favouring superior efficacy for PFA. We reported one case of cardiac tamponade in the PFA group, occurring during a procedure performed early in the center’s experience and attributed to catheter manipulation. Procedure time was shorter with PFA, and although not statistically significant, it was associated with a lower rate of AF recurrence compared to HPSD. Specifically, the PFA group exhibited significantly fewer AF recurrences when PWI was performed, as opposed to the group treated with HPSD ablation. While still undergoing initial real-world assessment, both methods have demonstrated effectiveness in achieving minimal AF recurrence during follow-up.
